# 1-Phenyl-1*H*-pyrazole-4-carbaldehyde

**DOI:** 10.1107/S1600536812010896

**Published:** 2012-03-17

**Authors:** Abdullah M. Asiri, Hassan M. Faidallah, Tariq R. Sobahi, Seik Weng Ng, Edward R. T. Tiekink

**Affiliations:** a Chemistry Department, Faculty of Science, King Abdulaziz University, PO Box 80203, Jeddah, Saudi Arabia; b The Center of Excellence for Advanced Materials Research, King Abdulaziz University, Jeddah, PO Box 80203, Saudi Arabia; c Department of Chemistry, University of Malaya, 50603 Kuala Lumpur, Malaysia

## Abstract

In the title mol­ecule, C_10_H_8_N_2_O, the five- and six-membered rings form a dihedral angle of 10.14 (9)°. The aldehyde group is almost coplanar with the pyrazole ring to which it is connected [O—C—C—C torsion angle = −179.35 (17)°]. In the crystal, inversion dimers are linked by four C—H⋯O inter­actions as the carbonyl O atom accepts two such bonds. The dimeric aggregates are linked into supra­molecular layers in the *ac* plane by C—H⋯π and π–π [ring centroid(pyrrole)⋯ring centroid(phen­yl) = 3.8058 (10) Å] inter­actions.

## Related literature

For the anti-bacterial properties of pyrazole derivatives, see: Kane *et al.* (2003 ▶). For related structures, see: Asiri, Al-Youbi, *et al.* (2012 ▶); Asiri, Faidallah *et al.* (2012 ▶). For the synthesis, see: Vera-DiVaio *et al.* (2009 ▶).
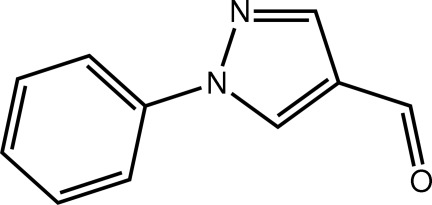


## Experimental

### 

#### Crystal data

C_10_H_8_N_2_O
*M* *_r_* = 172.18
Monoclinic, 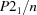

*a* = 11.1657 (10) Å
*b* = 5.0858 (4) Å
*c* = 15.3034 (11) Å
β = 111.130 (9)°
*V* = 810.60 (11) Å^3^
*Z* = 4
Mo *K*α radiation
μ = 0.10 mm^−1^
*T* = 100 K
0.30 × 0.30 × 0.15 mm

#### Data collection

Agilent SuperNova Dual diffractometer with an Atlas detector
Absorption correction: multi-scan (*CrysAlis PRO*; Agilent, 2011 ▶) *T*_min_ = 0.972, *T*_max_ = 0.986
3485 measured reflections
1814 independent reflections
1359 reflections with *I* > 2σ(*I*)
*R*_int_ = 0.050

#### Refinement

*R*[*F* ^2^ > 2σ(*F* ^2^)] = 0.050
*wR*(*F* ^2^) = 0.136
*S* = 0.99
1814 reflections
151 parameters
All H-atom parameters refined
Δρ_max_ = 0.28 e Å^−3^
Δρ_min_ = −0.26 e Å^−3^

### 

Data collection: *CrysAlis PRO* (Agilent, 2011 ▶); cell refinement: *CrysAlis PRO*; data reduction: *CrysAlis PRO*; program(s) used to solve structure: *SHELXS97* (Sheldrick, 2008 ▶); program(s) used to refine structure: *SHELXL97* (Sheldrick, 2008 ▶); molecular graphics: *ORTEP-3* (Farrugia, 1997 ▶) and *DIAMOND* (Brandenburg, 2006 ▶); software used to prepare material for publication: *publCIF* (Westrip, 2010 ▶).

## Supplementary Material

Crystal structure: contains datablock(s) global, I. DOI: 10.1107/S1600536812010896/bt5843sup1.cif

Structure factors: contains datablock(s) I. DOI: 10.1107/S1600536812010896/bt5843Isup2.hkl

Additional supplementary materials:  crystallographic information; 3D view; checkCIF report

## Figures and Tables

**Table 1:** Hydrogen-bond geometry (Å, °) *Cg*1 is the centroid of the C5–C10 ring.

*D*—H⋯*A*	*D*—H	H⋯*A*	*D*⋯*A*	*D*—H⋯*A*
C4—H4⋯O1^i^	0.960 (19)	2.432 (19)	3.379 (2)	168.6 (13)
C10—H10⋯O1^i^	0.978 (19)	2.335 (19)	3.303 (2)	170.8 (16)
C8—H8⋯*Cg*1^ii^	0.978 (18)	2.947 (18)	3.761 (2)	141.4 (14)

Symmetry codes: (i) 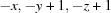; (ii) 
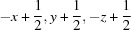
.

**Fig. 1.**
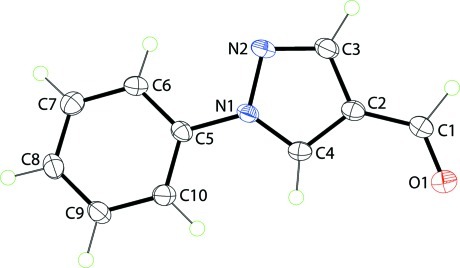
The molecular structure of (I) showing the atom-labelling scheme and displacement ellipsoids at the 50% probability level.

**Fig. 2.**
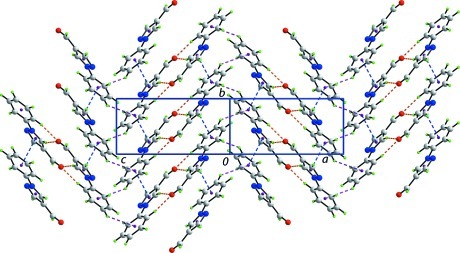
A view of the supramolecular layer in the ac plane in (I). The C—H···O, C—H···π and π—π interactions are shown as orange, purple and blue dashed lines, respectively.

**Fig. 3.**
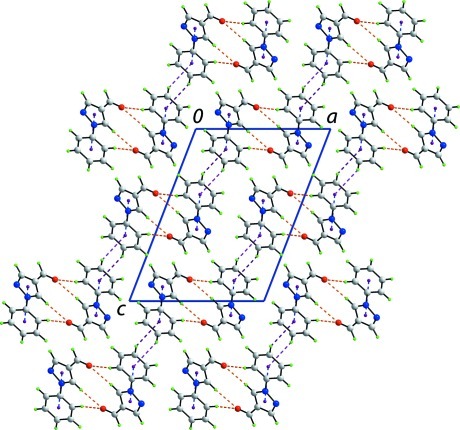
A view in projection down the b axis of the unit-cell contents of (I). The C—H···O, C—H···π and π—π interactions are shown as orange, purple and blue dashed lines, respectively.

## supplementary crystallographic information

### Comment

In continuation of structural studies of pyrazole derivatives (Asiri, Al-Youbi, *et al.*, 2012; Asiri, Faidallah *et al.*, 2012), of interest, for example, owing to their anti-bacterial activity (Kane *et al.*, 2003), the title compound, 1-phenyl-1*H*-pyrazole-4-carbaldehyde (I), was investigated crystallographically.

In (I), Fig. 1, the dihedral angle between the five- and six-membered rings is 10.14 (9) °, indicating a slight twist in the molecule. The aldehyde group is co-planar with the pyrazole ring to which it is connected as seen in the value of the O1—C1—C2—C3 torsion angle of -179.35 (17)°.

Inversion related molecules are connected into dimers *via* C—H···O interactions involving a bifurcated carbonyl-O atom, Table 1. Dimers are linked into supramolecular layers in the *ac* plane *via* C—H···π and π—π interactions occurring between the five- and six-membered rings [ring centroid···ring centroid distance = 3.8058 (10) Å, angle of inclination = 10.14 (9)° for symmetry operation: *x*, -1 + *y*, *z*], Fig. 2 and Table 1. Layers stack with no specific intermolecular interactions between them, Fig. 3.

### Experimental

*N*,*N*-Dimethylformamide (25.6 ml, 0.33 mmol) was stirred in around flask within an ice-bath and POCl_3_ (21.6 ml, 0.23 mmol) was added drop-wise. Then *N*-phenylpyrazole was added (4.4 ml, 0.033 mmol) to this cold mixture. The reaction was allowed to warm to room temperature and then heated at reflux for 6 h. The temperature was kept at 368–373 K. When the reaction was completed, the contents were poured onto crushed ice and made weakly alkaline with a saturated solution of sodium carbonate. The solid was filtered off, washed with water and recrystallized from ethanol. Yield: 65%. *M*.pt. 358–359 K. (lit. 358 K; Vera-DiVaio *et al.*, 2009).

### Refinement

H-atoms were freely refined; the range of C—H bond lengths is 0.960 (17)–1.023 (18) Å. Owing to poor agreement, the (3 2 6) reflection was omitted from the final cycles of refinement.

### Crystal data

**Table:**

C_10_H_8_N_2_O	*F*(000) = 360
*M**_r_* = 172.18	*D*_x_ = 1.411 Mg m^−^^3^
Monoclinic, *P*2_1_/*n*	Mo *K*α radiation, λ = 0.71073 Å
Hall symbol: -P 2yn	Cell parameters from 1251 reflections
*a* = 11.1657 (10) Å	θ = 2.8–27.5°
*b* = 5.0858 (4) Å	µ = 0.10 mm^−^^1^
*c* = 15.3034 (11) Å	*T* = 100 K
β = 111.130 (9)°	Prism, light-brown
*V* = 810.60 (11) Å^3^	0.30 × 0.30 × 0.15 mm
*Z* = 4	

### Data collection

**Table:**

Agilent SuperNova Dual diffractometer with an Atlas detector	1814 independent reflections
Radiation source: SuperNova (Mo) X-ray Source	1359 reflections with *I* > 2σ(*I*)
Mirror monochromator	*R*_int_ = 0.050
Detector resolution: 10.4041 pixels mm^-1^	θ_max_ = 27.6°, θ_min_ = 2.8°
ω scan	*h* = −14→14
Absorption correction: multi-scan (*CrysAlis PRO*; Agilent, 2011)	*k* = −5→6
*T*_min_ = 0.972, *T*_max_ = 0.986	*l* = −19→13
3485 measured reflections	

### Refinement

**Table:**

Refinement on *F*^2^	Secondary atom site location: difference Fourier map
Least-squares matrix: full	Hydrogen site location: inferred from neighbouring sites
*R*[*F*^2^ > 2σ(*F*^2^)] = 0.050	All H-atom parameters refined
*wR*(*F*^2^) = 0.136	*w* = 1/[σ^2^(*F*_o_^2^) + (0.0732*P*)^2^] where *P* = (*F*_o_^2^ + 2*F*_c_^2^)/3
*S* = 0.99	(Δ/σ)_max_ = 0.001
1814 reflections	Δρ_max_ = 0.28 e Å^−^^3^
151 parameters	Δρ_min_ = −0.26 e Å^−^^3^
0 restraints	Extinction correction: *SHELXL97* (Sheldrick, 2008), Fc^*^=kFc[1+0.001xFc^2^λ^3^/sin(2θ)]^-1/4^
Primary atom site location: structure-invariant direct methods	Extinction coefficient: 0.013 (4)

### Fractional atomic coordinates and isotropic or equivalent isotropic displacement parameters (Å^2^)

**Table:**

	*x*	*y*	*z*	*U*_iso_*/*U*_eq_	
O1	0.11384 (11)	0.2706 (2)	0.62516 (8)	0.0286 (4)	
N1	0.26714 (12)	0.9228 (3)	0.52304 (9)	0.0196 (3)	
N2	0.38346 (13)	0.9268 (3)	0.59584 (9)	0.0247 (4)	
C1	0.21644 (15)	0.3842 (3)	0.65682 (11)	0.0230 (4)	
C2	0.25665 (15)	0.6026 (3)	0.61478 (11)	0.0209 (4)	
C3	0.37465 (16)	0.7329 (3)	0.65005 (12)	0.0245 (4)	
C4	0.18990 (15)	0.7336 (3)	0.53228 (11)	0.0204 (4)	
C5	0.24267 (15)	1.1131 (3)	0.45059 (10)	0.0196 (4)	
C6	0.34207 (16)	1.2698 (3)	0.44624 (11)	0.0226 (4)	
C7	0.31714 (17)	1.4569 (4)	0.37669 (12)	0.0248 (4)	
C8	0.19463 (17)	1.4863 (4)	0.31105 (11)	0.0247 (4)	
C9	0.09706 (17)	1.3272 (4)	0.31553 (12)	0.0263 (4)	
C10	0.11990 (16)	1.1412 (3)	0.38573 (12)	0.0242 (4)	
H1	0.2818 (16)	0.327 (4)	0.7201 (13)	0.022 (4)*	
H3	0.4456 (17)	0.689 (4)	0.7089 (13)	0.027 (5)*	
H4	0.1060 (17)	0.707 (3)	0.4858 (12)	0.022 (5)*	
H6	0.4267 (19)	1.239 (4)	0.4903 (14)	0.033 (5)*	
H7	0.3846 (16)	1.567 (4)	0.3715 (12)	0.023 (5)*	
H8	0.1801 (15)	1.619 (4)	0.2621 (12)	0.019 (4)*	
H9	0.0057 (18)	1.351 (4)	0.2696 (14)	0.037 (5)*	
H10	0.0500 (15)	1.030 (4)	0.3886 (12)	0.023 (5)*	

### Atomic displacement parameters (Å^2^)

**Table:**

	*U*^11^	*U*^22^	*U*^33^	*U*^12^	*U*^13^	*U*^23^
O1	0.0250 (7)	0.0306 (8)	0.0274 (6)	−0.0045 (5)	0.0063 (5)	−0.0006 (6)
N1	0.0183 (7)	0.0214 (8)	0.0154 (6)	0.0002 (5)	0.0015 (5)	−0.0005 (6)
N2	0.0193 (7)	0.0286 (8)	0.0194 (7)	−0.0011 (6)	−0.0013 (6)	0.0005 (6)
C1	0.0229 (9)	0.0254 (9)	0.0184 (8)	0.0017 (7)	0.0046 (7)	−0.0011 (7)
C2	0.0205 (8)	0.0216 (9)	0.0186 (7)	0.0007 (6)	0.0047 (6)	−0.0032 (7)
C3	0.0225 (9)	0.0270 (10)	0.0192 (8)	0.0005 (7)	0.0018 (7)	0.0001 (7)
C4	0.0182 (8)	0.0220 (9)	0.0185 (7)	−0.0007 (6)	0.0035 (7)	−0.0038 (7)
C5	0.0230 (8)	0.0197 (9)	0.0142 (7)	0.0011 (6)	0.0042 (6)	−0.0017 (6)
C6	0.0196 (9)	0.0263 (10)	0.0191 (8)	−0.0015 (7)	0.0038 (7)	−0.0026 (7)
C7	0.0271 (9)	0.0243 (9)	0.0238 (8)	−0.0024 (7)	0.0103 (7)	−0.0034 (8)
C8	0.0313 (9)	0.0205 (9)	0.0209 (8)	0.0021 (7)	0.0076 (7)	0.0027 (8)
C9	0.0242 (9)	0.0253 (10)	0.0236 (8)	0.0031 (7)	0.0017 (7)	0.0037 (8)
C10	0.0212 (9)	0.0222 (9)	0.0251 (8)	−0.0010 (7)	0.0034 (7)	0.0002 (8)

### Geometric parameters (Å, º)

**Table:**

O1—C1	1.2168 (19)	C5—C10	1.380 (2)
N1—C4	1.333 (2)	C5—C6	1.387 (2)
N1—N2	1.3731 (17)	C6—C7	1.379 (2)
N1—C5	1.422 (2)	C6—H6	0.96 (2)
N2—C3	1.315 (2)	C7—C8	1.383 (2)
C1—C2	1.435 (2)	C7—H7	0.963 (18)
C1—H1	1.023 (18)	C8—C9	1.379 (3)
C2—C4	1.384 (2)	C8—H8	0.978 (18)
C2—C3	1.398 (2)	C9—C10	1.384 (2)
C3—H3	0.986 (18)	C9—H9	1.016 (18)
C4—H4	0.960 (17)	C10—H10	0.977 (17)
			
C4—N1—N2	112.57 (12)	C10—C5—N1	119.55 (14)
C4—N1—C5	128.61 (13)	C6—C5—N1	119.75 (14)
N2—N1—C5	118.81 (13)	C7—C6—C5	119.40 (15)
C3—N2—N1	103.68 (13)	C7—C6—H6	122.1 (12)
O1—C1—C2	126.22 (15)	C5—C6—H6	118.4 (12)
O1—C1—H1	119.3 (10)	C6—C7—C8	120.47 (16)
C2—C1—H1	114.5 (10)	C6—C7—H7	121.1 (11)
C4—C2—C3	104.28 (15)	C8—C7—H7	118.4 (11)
C4—C2—C1	129.01 (15)	C9—C8—C7	119.52 (17)
C3—C2—C1	126.71 (15)	C9—C8—H8	121.9 (10)
N2—C3—C2	112.73 (14)	C7—C8—H8	118.6 (10)
N2—C3—H3	121.7 (11)	C8—C9—C10	120.80 (16)
C2—C3—H3	125.6 (11)	C8—C9—H9	120.6 (11)
N1—C4—C2	106.74 (14)	C10—C9—H9	118.5 (11)
N1—C4—H4	121.3 (10)	C5—C10—C9	119.10 (16)
C2—C4—H4	131.9 (11)	C5—C10—H10	120.7 (10)
C10—C5—C6	120.70 (15)	C9—C10—H10	120.2 (10)
			
C4—N1—N2—C3	0.04 (18)	N2—N1—C5—C10	−169.04 (14)
C5—N1—N2—C3	179.12 (14)	C4—N1—C5—C6	−170.62 (15)
O1—C1—C2—C4	1.2 (3)	N2—N1—C5—C6	10.5 (2)
O1—C1—C2—C3	−179.35 (17)	C10—C5—C6—C7	0.6 (2)
N1—N2—C3—C2	0.16 (19)	N1—C5—C6—C7	−178.94 (14)
C4—C2—C3—N2	−0.3 (2)	C5—C6—C7—C8	−0.8 (3)
C1—C2—C3—N2	−179.83 (16)	C6—C7—C8—C9	0.0 (3)
N2—N1—C4—C2	−0.21 (18)	C7—C8—C9—C10	1.0 (3)
C5—N1—C4—C2	−179.18 (15)	C6—C5—C10—C9	0.4 (3)
C3—C2—C4—N1	0.29 (17)	N1—C5—C10—C9	179.95 (15)
C1—C2—C4—N1	179.81 (16)	C8—C9—C10—C5	−1.2 (3)
C4—N1—C5—C10	9.9 (3)		

### Hydrogen-bond geometry (Å, º)

Cg1 is the centroid of the C5–C10 ring.

**Table:**

*D*—H···*A*	*D*—H	H···*A*	*D*···*A*	*D*—H···*A*
C4—H4···O1^i^	0.960 (19)	2.432 (19)	3.379 (2)	168.6 (13)
C10—H10···O1^i^	0.978 (19)	2.335 (19)	3.303 (2)	170.8 (16)
C8—H8···*Cg*1^ii^	0.978 (18)	2.947 (18)	3.761 (2)	141.4 (14)

Symmetry codes: (i) −*x*, −*y*+1, −*z*+1; (ii) −*x*+1/2, *y*+1/2, −*z*+1/2.
